# Evaluation of Lipid Profile, High-Sensitivity C-reactive Protein (hs-CRP), and Homocysteine in Premature Acute Myocardial Infarction in a Semi-urban Population: A Case-Control Study

**DOI:** 10.7759/cureus.109152

**Published:** 2026-05-18

**Authors:** Monika N Chavan, Anup N Nillawar, Anup S Hendre, Axita Vani, Satish Kakade, Abhijeet Shelke

**Affiliations:** 1 Department of Biochemistry, Krishna Institute of Medical Sciences, Krishna Vishwa Vidyapeeth (Deemed to be University), Karad, IND; 2 Department of Biochemistry, BKL Walawalkar Rural Medical College, Chiplun, IND; 3 Department of Community Medicine, Krishna Institute of Medical Sciences, Krishna Vishwa Vidyapeeth (Deemed to be University), Karad, IND; 4 Department of Cardiology, Krishna Institute of Medical Sciences, Krishna Vishwa Vidyapeeth (Deemed to be University), Karad, IND

**Keywords:** dyslipidemia, high-sensitivity c-reactive protein (hs-crp), homocysteine, premature acute myocardial infarction, young adults

## Abstract

Background

The incidence of acute myocardial infarction (AMI) in young adults is progressively increasing, presenting a significant public health challenge. While traditional risk factors are documented, specific roles of atherogenic dyslipidemia and novel inflammatory biomarkers in premature AMI remain under-evaluated in semi-urban populations. Recent evidence suggests that biomarkers such as homocysteine (Hcy) and high-sensitivity C-reactive protein (hs-CRP) indicate endothelial dysfunction and vascular inflammation. Hyperhomocysteinemia (HHcy) is associated with oxidative stress and endothelial damage, while elevated hs-CRP levels are linked to plaque instability and a higher risk of cardiovascular events.

Aims

To evaluate the serum lipid profile, hs-CRP, and Hcy levels in patients with premature AMI in a semi-urban population, and to assess their correlations with glycemic markers.

Methods

A case-control analytical study was conducted at a tertiary care center in Maharashtra, India. The study enrolled 105 participants divided into three groups: Healthy controls (n=53), old age AMI (≥40 years, n=32), and premature AMI (<40 years, n=20). Fasting venous blood was analyzed for lipid profile, hs-CRP, Hcy, and glycemic markers.

Results

Patients with premature AMI were predominantly male (85%) with high rates of physical inactivity (90%). This cohort demonstrated profound atherogenic dyslipidemia, characterized by significantly elevated triglycerides (TGs) (238.00 ± 118.75 mg/dL). In premature AMI, TGs showed a significant positive correlation with fasting blood sugar (FBS) (r=0.681, p≤ 0.001), and glycated hemoglobin (HbA1c) (r=0.367, p=0.039). Inflammatory markers were also markedly elevated, with hs-CRP strongly correlating with FBS (r=0.658, p≤0.001). Older patients with AMI showed no such significant correlation between TGs and glycemic markers.

Conclusions

Premature AMI in this semi-urban population is metabolically distinct, driven by isolated hypertriglyceridemia and chronic inflammation linked to subclinical insulin resistance. A combined assessment of the lipid profile, hs-CRP, and Hcy levels may improve early risk stratification by capturing both atherogenic and inflammatory pathways. Early targeted screening for these biomarkers is crucial for prevention.

## Introduction

Acute myocardial infarction (AMI) is one of the major causes of morbidity and mortality around the world [1] despite significant progress in preventive and therapeutic strategies. The most dangerous type of coronary heart disease (CHD) is myocardial infarction (MI), which may sometimes lead to sudden death. Although it is most commonly observed in individuals over the age of 45 years, it can also occur in younger adults, both males and females. This shift highlights premature AMI as an emerging clinical phenotype, with unique risk factors, distinct pathways, and important implications for early detection and prevention [2].

In recent years, an alarming increase in the incidence of AMI among younger individuals has been noted, which is a significant public health issue [3]. Premature MI refers to a heart attack that occurs at a young age and is typically defined as an MI in men aged 18-55 years and in women aged 18-65 years [4]. Although broader epidemiological definitions exist, the present study operationally defined premature AMI as the occurrence before 40 years of age. This differentiation highlights the earlier onset of coronary artery disease (CAD) in men and is commonly used in clinical and epidemiological studies to identify early-onset cardiovascular events [5].

It is estimated that worldwide deaths in 2019 from cardiovascular diseases (CVDs) were 8.9 million. During the 10-year follow-up of the Framingham Heart Study, the incidence of AMI in men aged between 30 and 34 years was 12.9 per 1000, and it was 5.2 per 1000 in women aged between 35 and 44 years [6].

India has one of the highest rates of CVD worldwide. According to predictions, the annual number of deaths from CVD in India is projected to rise from 2.26 million in 1990 to nearly 4.77 million in 2020 [1,7]. Over the past few decades, the prevalence rates of CHD in India have been estimated to be 1.6% to 7.4% in rural areas and from 1% to 13.2% in urban populations [1]. The INTERHEART study revealed that, even at younger ages, Indians have higher rates of CVD risk factors compared to other ethnic groups, including diabetes, hypertension, and abdominal obesity [8]. Multiple risk factors are implicated in this rise, ranging from family history, cigarette smoking, hypertension, diabetes mellitus, and obesity to various psychosocial stresses imposed by social dynamics and urbanization [9].

The prevalence rate of CVD has significantly increased in India in the last 25 years. Over the last three decades, AMI prevalence increased from 1.1% to 7.5% in urban areas and 2.1% to 3.7% in rural populations. Despite this growing concern, larger data are not readily available regarding the specific phenotypic presentation of premature AMI in semi-urban populations [10].

AMI, commonly known as a heart attack, is caused by a sudden interruption of blood flow to the heart muscle, most often due to atherosclerotic plaque rupture and thrombus (blood clot) formation in a coronary artery. As a result, myocardial tissue is deprived of oxygen, which causes ischemic injury and cell death [11]. AMI is said to have occurred when there is a presence of acute myocardial injury detected by abnormal cardiac biomarkers in the setting of evidence of acute myocardial ischemia [12]. AMI is caused by both modifiable and non-modifiable risk factors, ranging from dyslipidemia, hypertension, diabetes mellitus, family history, and unhealthy behaviors such as cigarette smoking, alcohol consumption, physical inactivity, and poor dietary habits, which remain common worldwide [13].

High-sensitivity C-reactive protein (hs-CRP) reflects low-grade inflammation throughout the body [14]. Beyond abnormal lipid levels, hs-CRP is also recognized as an independent factor that contributes to CVD risk [15]. Evidence from clinical studies has shown that circulating hs-CRP concentrations are predictive of future clinical outcomes and are associated with infarct size in patients experiencing an AMI. These findings suggest that hs-CRP reflects both the severity of myocardial injury and the burden of coronary inflammation, thereby supporting its utility as a valuable biomarker for improved risk stratification in premature AMI [16].

Homocysteine (Hcy) is an established marker of endothelial injury and thrombotic risk in CAD, and was included in the present study to benchmark its diagnostic performance against hs-CRP in a premature AMI cohort.

Both Hcy and hs-CRP were measured to evaluate their comparative diagnostic performance as biomarkers for premature AMI. The present study limits its analysis to their discriminatory ability as assessed by the receiver operating characteristic (ROC) curve analysis; a detailed mechanistic investigation of hyperhomocysteinemia (HHcy) and its metabolic determinants in this population is the subject of a separate study currently underway.

While dyslipidemia is a known modifiable risk factor, and markers such as Hcy and hs-CRP indicate endothelial damage and inflammation, their combined interaction with early metabolic disturbances remains unclear in young adults. The goal of this case-control analytical study is to fill this knowledge gap. Specifically, this study evaluates whether an underlying TG-glucose axis and subclinical insulin resistance serve as primary pathophysiological mechanisms in premature AMI, distinguishing it from the pathophysiology seen in older patients with AMI.

## Materials and methods

Selection of study subjects

The study was a case-control analytical study conducted in the Department of Biochemistry, in collaboration with the Department of Cardiology at Krishna Vishwa Vidyapeeth, Karad, Maharashtra, India. A total of 105 individuals were enrolled in the study and divided into three groups: controls comprised two groups: control 1 (healthy individuals, n=53) and control 2 (old age adults with AMI, n=32), whereas the case group included patients with premature AMI (n=20). The control 1 group included healthy individuals (n=53) without any comorbidities, and the control 2 group contained old-age patients with AMI (n=32) admitted to the emergency department/cardiac care unit (CCU). The case group (premature AMI) consisted of young patients diagnosed with AMI (n=20).

Inclusion criteria

Control Group 1/(˗ve Controls)

Participants who were age- and sex-matched with cases of AMI visited the outpatient department (OPD) with non-specific generalized complaints and a normal electrocardiogram (ECG), and had no history or clinical/laboratory evidence of myocardial infarction (MI).

Control Group 2/(+ve Controls)

Participants who were age- and sex-mismatched with cases of AMI, age > 40 years with abnormal findings of ECG suggestive of AMI, along with increased creatine phosphokinase-MB (CPK-MB) level or troponin levels.

Cases

Individuals who were aged 20-40 years, and presented with abnormal findings of ECG suggestive of AMI and or increased CPK-MB level or troponin levels.

Exclusion criteria

Patients in the age groups < 20 years and > 70 years were excluded. Patients with active inflammation or infection, liver disease, tuberculosis, oncologic disease, uremia, thyroid disorders or other endocrine diseases, pregnant women and lactating mothers, and individuals who had consumed multivitamin supplements in the last three months were excluded. Additionally, patients receiving lipid-lowering drugs or anti-inflammatory medications were excluded to minimize potential confounding effects.

The diagnosis of patients with AMI admitted to the CCU was established according to three clinical criteria, including cardiac troponins and CPK activity, ECG changes, and associated clinical symptoms.

The patient selection was conducted according to inclusion and exclusion criteria using the purposive sampling method. After the explanation of the study, written informed consent was obtained from each participant. Preliminary information about age, diet, clinical complications, duration of MI, and family history was collected with the help of a questionnaire (Proforma).

This study was approved by the institutional Ethics Committee (Ref. No. KVV/ IEC/09/2023 Protocol no. 456/2022-2023).

Collection and processing of blood samples

Fasting venous blood samples were collected from the study and control participants following an 8-12-hour overnight fast. These samples were analyzed for fasting blood sugar (FBS), lipid profile (total cholesterol (TC), triglycerides (TGs), high‑ and low‑density lipoprotein cholesterol), Hcy, hs-CRP, and glycated hemoglobin (HbA1c). The collected venous blood samples were centrifuged at 3,000 rpm for 5 minutes and stored at ‑20°C until being analyzed. All biochemical parameters were analyzed within 1-2 weeks of sample collection to ensure biomarker stability. The following parameters were estimated: serum lipid profile using a colorimetric method (Abbott Architect C4000; Abbott Laboratories, Abbott Park, Illinois, USA), serum Hcy using the chemiluminescent microparticle immunoassay (CMIA) method (Abbott Architect i1000SR; Abbott Laboratories, Abbott Park, Illinois, USA), hs-CRP using the turbidimetric/immunoturbidimetric method (Abbott Architect C4000; Abbott Laboratories, Abbott Park, Illinois, USA), FBS using the enzymatic method (Abbott C4000), and HbA1c measured using the Bio-Rad D-10 high-performance liquid chromatography (HPLC; Bio-Rad Laboratories, Hercules, California, USA).

Statistical analysis

The data were analyzed using SPSS Statistics for Windows, version 20 (released 2011; IBM Corp., Armonk, NY, USA). The results are expressed as mean ± standard deviation. The Chi-square test and analysis of variance (ANOVA) test were used for comparison of the groups. The relationship between variables was determined using Pearson’s correlation coefficient. A difference was considered statistically significant when the p-value was ≤ 0.05. Additionally, the area under the curve (AUC), sensitivity, and specificity were measured.

## Results

Table 1 represents baseline and anthropometric data in the three groups, as defined earlier, with a total of 105 patients. The groups are as follows: healthy controls (control 1; n=53), older patients with AMI (control 2; n=32), and young patients with AMI (premature AMI) (cases; n=20).

**Table 1:** Demographic profile of study subjects. BMI: body mass index; SD: standard deviation **p < 0.01

Sr. no.	Variables	Control 1 healthy (n=53) (mean ± SD)	Control 2 old-age AMI (n=32) (mean ± SD)	Cases premature AMI (n=20) (mean ± SD)
1	Age (years)	34.96 ± 5.06**	57.03 ± 8.43**	35.65 ± 4.35**
2	BMI (kg/m^2^)	23.86 ± 2.46**	24.61 ± 3.97	28.09 ± 5.37**

The mean age (± standard deviation [SD]) of healthy controls was 34.96 ± 5.06 years, whereas the mean age of participants with old-age AMI was significantly higher, 57.03 ± 8.43 years (p<0.01). The mean age of the premature AMI group was 35.65 ± 4.35 years (p<0.01). No significant age difference was observed between the healthy controls and the premature AMI group.

Body mass index (BMI) varied significantly across the groups (p<0.01), with both AMI groups having higher BMI values, especially in the premature AMI group (28.09 ± 5.37), followed by the old age AMI group (24.61 ± 3.97), compared to healthy controls (23.86 ± 2.46). Our findings represent the clear age- and BMI-related variations among the study groups, highlighting a burden of overweight and obesity among patients with premature AMI (Table 1).

Table 2 shows the baseline sociodemographic and lifestyle characteristics in the three groups, as defined earlier, with a total of 105 patients.

**Table 2:** Baseline sociodemographic and lifestyle characteristics among study groups. AMI: acute myocardial infarction; CAD: coronary artery disease

Sr. no.	Variables		Control 1 healthy (n=53) n (%)	Control 2 old-age AMI (n=32) n (%)	Cases premature AMI (n=20) n (%)
1	Gender	Male	34 (64.2)	21 (65.6)	17 (85)
		Female	19 (35.8)	11 (34.4)	3 (15)
2	Background	Rural	17 (32)	26 (81.2)	14( 70)
		Urban	36 (68)	6 (18.8)	6 (30)
3	Smoking status	Yes	6 (11.3)	3 (9.4)	6 (30)
		No	47 (88.7)	29 (90.6)	14 (70)
4	Tobacco consumption	Yes	0 (0)	17 (53.1)	0 (0)
		No	53 (100)	15 (46.9)	20 (100)
5	Alcohol consumption	Yes	12 (22.6)	6 (18.8)	11 (55)
		No	41 (77.4)	26 (81.2)	9 (45)
6	Physical activity	Yes	36 (68)	28 (87.5)	2 (10)
		No	17 (32)	4 (12.5)	18 (90)
7	Dietary habit	Pure vegetarian	6 (11.3)	2 (6.2)	0 (0)
		Mixed	44 (83)	26 (81.3)	3 (15)
		Mixed + highly non-vegetarian	3 (5.7)	4 (12.5)	17 (85)
		Vegan	0 (0)	0 (0)	0 (0)
8	Family history of CAD	Yes	0 (0)	4 (12.5)	2 (10)
		No	53 (100)	28 (87.5)	18 (90)

In our study, the male population was more common than the female across all study groups. In the healthy control group, 64.2% were male. A similar pattern was observed in the old-age AMI group, where 65.6% were men, and the imbalance was most evident in the premature AMI group, in which the male proportion was the highest (85%). Correspondingly, the representation of female participants was a smaller proportion in each group, with the premature AMI group having the lowest proportion of females at just 15%.

Looking for residential status, the majority of healthy controls were from urban areas (67.9%). In contrast, most patients with AMI were predominantly from rural/semi-urban backgrounds, which was noted among old-age patients with AMI (81.2%) and those with premature AMI (70%), indicating a higher occurrence of AMI among individuals from rural/semi-urban settings in both groups.

Premature patients with AMI showed a higher percentage of smoking, alcohol use, and physical inactivity compared to other groups, whereas tobacco use was only observed in older patients with AMI. Dietary patterns also differed across the groups. Young patients with AMI consumed mostly a mixed diet with a high proportion of a non-vegetarian diet (85%), predominantly red meat consumption.

However, the least chances of family history of CAD were observed in old-age AMI (12.5%) and premature AMI groups (10%) (Table 2).

Table 3 represents the comparative distribution of electrocardiographically confirmed AMI types between old-age AMI (control 2 group; n=32) and premature AMI groups (n=20). Anterior wall myocardial infarction (AWMI) was found most frequently in both groups; it was observed in 19 cases (59.4%) in old-age AMI and 10 cases (50%) in premature AMI. The second most common presentation observed in both groups was inferior wall MI (IWMI), occurring in nine older patients (28.1%) and eight young patients with AMI (40%). Lateral wall MI (LWMI) was observed in a smaller proportion compared to other types in both groups, and documented in four old-age patients with AMI (12.5%) and two premature patients with AMI (10%). Overall, anterior wall AMI predominated across both groups, with only modest variations in the distribution of IWMI and LWMI.

**Table 3:** Comparative distribution of ECG-confirmed myocardial infarction types across study groups. AMI: acute myocardial infarction; AWMI: anterior wall myocardial infarction; IWMI: inferior wall myocardial infarction; LWMI: lateral wall myocardial infarction; ECG: electrocardiograph

Sr. no.	ECG findings	Control 2 old-age AMI (n = 32) n (%)	Cases premature AMI (n = 20) n (%)
1	AWMI	19 (59.4)	10 (50)
2	IWMI	09 (28.1)	08 (40)
3	LWMI	04 (12.5)	02 (10)

Table 4 shows significant alterations in biochemical parameters among the study groups (controls, premature AMI, and old-age AMI).

**Table 4:** Comparison of biochemical parameters among the study groups. hs-CRP: high-sensitivity C-reactive protein, HbA1c: glycated hemoglobin; SD: standard deviation; AMI: acute myocardial infarction *p < 0.05, **p < 0.01

Parameters	Control 1 healthy (n=53) (mean ± SD)	Control 2 old-age AMI (n=32) (mean ± SD)	Cases premature AMI (n=20) (mean ± SD)
hs-CRP (mg/L)	0.196 ± 0.169*	6.02 ± 4.72*	4.33 ± 4.99**
Homocysteine (µmol/L)	9.26 ± 2.71	19.78 ± 12.79	17.54 ± 10.93
Fasting blood sugar (mg/dL)	85.36 ± 19.22	96.78 ± 27.94**	143.46 ± 84.81**
HbA1C (%)	4.30 ± 0.14	4.33 ± 0.37**	4.51 ± 0.26**

Significant alterations in metabolic and inflammatory parameters were observed in the AMI groups. Both AMI groups showed higher average levels of Hcy, hs-CRP, and HbA1c. Both old-age patients with AMI (6.02 ± 4.72 mg/L) and patients with premature AMI (4.33 ± 4.99 mg/L) had significantly elevated serum hs-CRP levels (p<0.01) compared to healthy controls (0.196 ± 0.169 mg/L), indicating severe systemic inflammation in AMI cases. Hcy concentrations were elevated in old-age AMI (19.78 ± 12.79 µmol/L) and premature AMI groups (17.54 ± 10.93 µmol/L) compared to healthy participants (9.26 ± 2.71 µmol/L).

Patients with MI had significantly higher FBS levels than controls. The premature AMI group had the highest values (143.46 ± 84.81 mg/dL), followed by old-age patients with AMI (96.78 ± 27.94 mg/dL) (p<0.01) when compared with normal healthy controls (85.36 ± 19.22 mg/dL). Although HbA1c values differed statistically among groups, mean values stayed within the non-diabetic range (Table 4).

**Figure 1:**
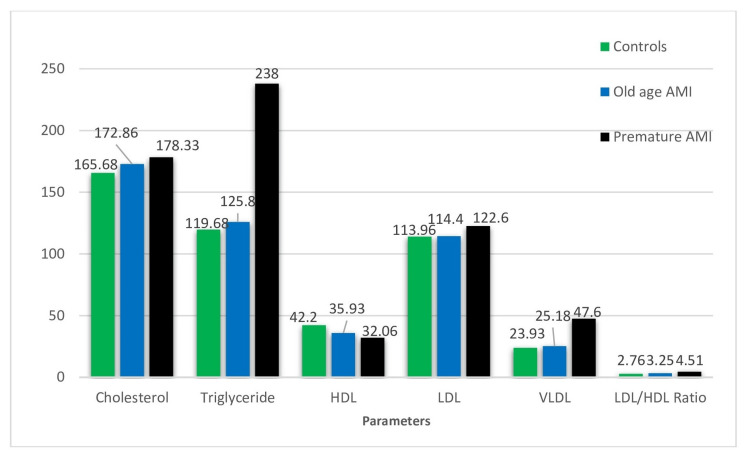
Comparison of lipid profile between the study groups. AMI: acute myocardial infarction; LDL: low-density lipoprotein; HDL: high-density lipoprotein; VLDL: very low-density lipoprotein

Table 5 and Figure 1 show a comparison of lipid profiles between the study groups. The AMI groups showed higher average levels of TC, TGs, low-density lipoproteins (LDL), and LDL/high-density lipoprotein (HDL) ratio, while exhibiting lower HDLs compared to the control group. The analysis revealed a statistically significant alteration in the lipid profiles of both premature and old-age patients with AMI when compared with the control group.

**Table 5:** Comparison of lipid profile parameters among the study groups. LDL: low-density lipoprotein; HDL: high-density lipoprotein; VLDL: very low-density lipoprotein; SD: standard deviation; AMI: acute myocardial infarction *p < 0.05; ** p < 0.01

Parameters	Control 1 healthy (n=53) (mean ± SD)	Control 2 old-age AMI (n=32) (mean ± SD)	Cases premature AMI (n=20) (mean ± SD)
Cholesterol (mg/dL)	165.68 ± 29.59	172.86 ± 42.35*	178.33 ± 53.25**
Triglyceride (TG) (mg/dL)	119.68 ± 62.37**	125.80 ± 48.23 **	238.00 ± 118.75**
HDL (mg/dL)	42.20 ± 7.13	35.93 ± 7.35*	32.06 ± 10.02**
LDL (mg/dL)	113.96 ± 18.78*	114.40 ± 27.44*	122.60 ± 41.58 **
VLDL (mg/dL)	23.93 ± 12.47**	25.18 ± 9.64**	47.60 ± 23.75**
LDL/HDL ratio	2.76 ± 0.70*	3.25 ± 0.92*	4.51 ± 3.20**

A one-way ANOVA was performed for each lipid parameter, followed by Tukey’s honestly significant difference (HSD) post-hoc test for pairwise group comparisons. A p-value of <0.05 was considered statistically significant.

The dominant lipid phenotype observed was atherogenic dyslipidemia rather than isolated hypercholesterolemia, characterized primarily by elevated triglycerides (TG) and reduced high-density lipoprotein (HDL) cholesterol levels rather than increased low-density lipoprotein (LDL) burden (Table 5, Figure 1).

As shown in Table 5, significant differences were observed among the study groups for all lipid parameters (p<0.001). Total cholesterol (TC), triglycerides (TG), LDL cholesterol, and very low-density lipoprotein (VLDL) cholesterol levels were significantly higher in both the premature and older AMI groups than in controls. Premature AMI patients demonstrated markedly elevated TG levels. In contrast, HDL cholesterol levels were significantly lower in both AMI groups than in the control group.

Correlation of metabolic and inflammatory parameters

To further understand the metabolic phenotype of premature AMI, Pearson's correlation coefficient was utilized. A striking, age-specific pathophysiological pattern emerged. In the premature AMI group, TGs were significantly and positively correlated with both FBS (r=0.681, p≤0.001) and HbA1c (r=0.367, p=0.039). Uniquely in the young cohort, the severity of inflammation was tightly coupled with glycemic dysregulation; hs-CRP showed a strong, significant positive correlation with FBS (r=0.658, p≤0.001) and HbA1c (r=0.373, p=0.036). Conversely, older patients with AMI showed no significant correlation between TGs and glycemic markers (TG/FBS, r=0.270 and p=0.331; TG/HbA1c, r=0.142 and p=0.430).

Receiver operating characteristic (ROC) curve for homocysteine and hs-CRP in premature AMI

**Figure 2:**
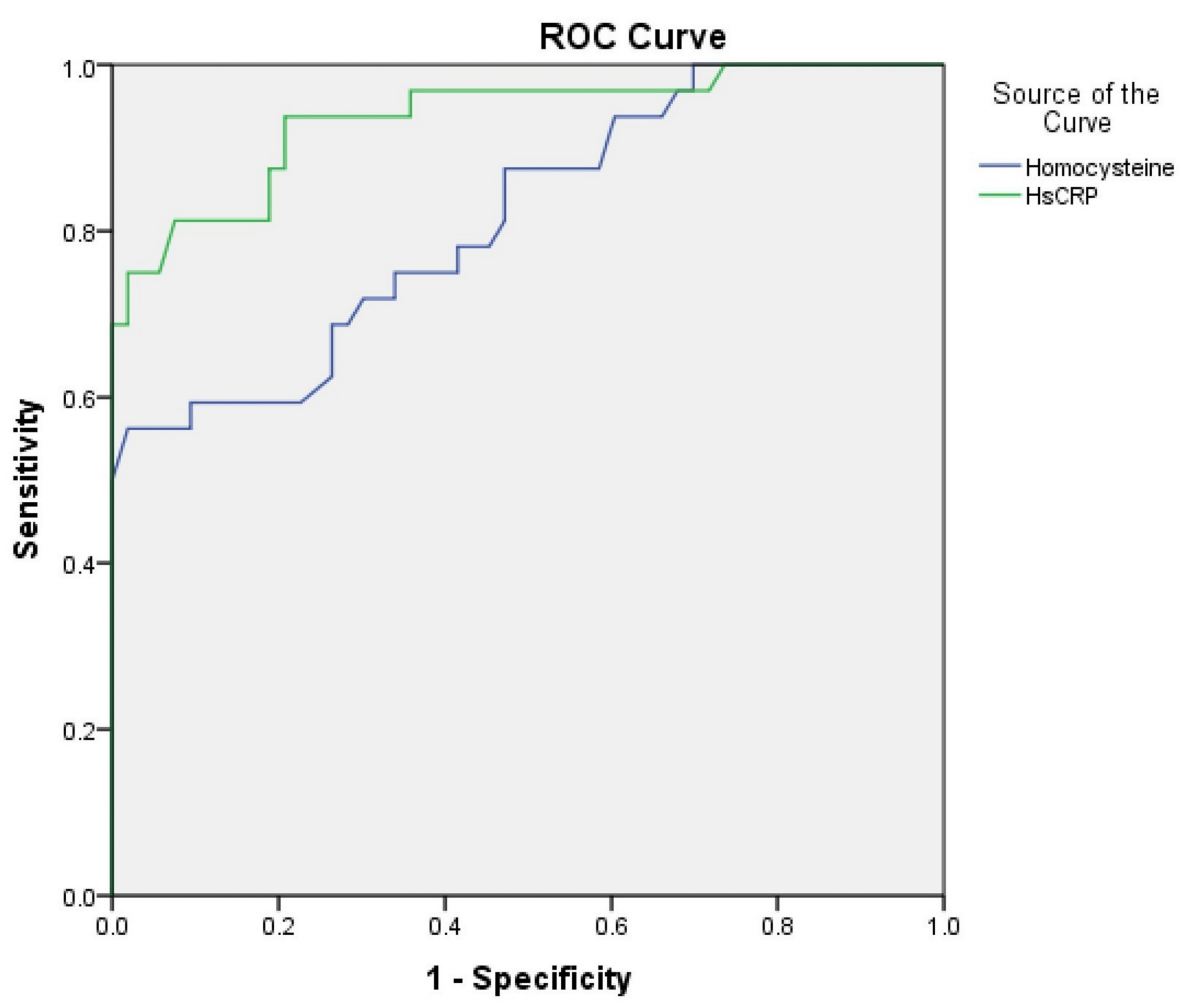
ROC curve for homocysteine and hs-CRP ROC: receiver operating characteristic; hs-CRP: high-sensitivity C-reactive protein

The ROC analysis was used to evaluate how well each marker distinguished between premature patients with AMI and healthy individuals. The AUC is a measure of this ability, with 1.0 being a perfect score. AUC for Hcy was 0.816 (p<0.001), and that for hs-CRP was 0.936 (p<0.001), as shown in Figure 2. hs-CRP had a slightly higher AUC (0.936 vs. 0.816), suggesting it is a marginally better primary stratification marker for identifying MI (Table 6).

**Table 6:** Receiver operating characteristic curve analysis of Premature AMI AUC: area under the curve, hs-CRP: high-sensitivity C-reactive protein

Variables	AUC	p-value	95% confidence interval (CI)		Sensitivity	Specificity	Accuracy
			Lower bound	Upper bound			
Homocysteine	0.816	0.001	0.721	0.910	71.9%	69.8%	70.6%
hs-CRP	0.936	0.001	0.880	0.992	93.8%	79.2%	84.7%

ROC curve for Hcy and hs-CRP in old-age AMI

The ROC analysis was used to evaluate how well each marker distinguished between old age patients with AMI and healthy individuals. The AUC is a measure of this ability, with 1.0 being a perfect score. The AUC for Hcy was 0.698 (p<0.001), and that for hs-CRP was 0.851 (p<0.001).

**Figure 3:**
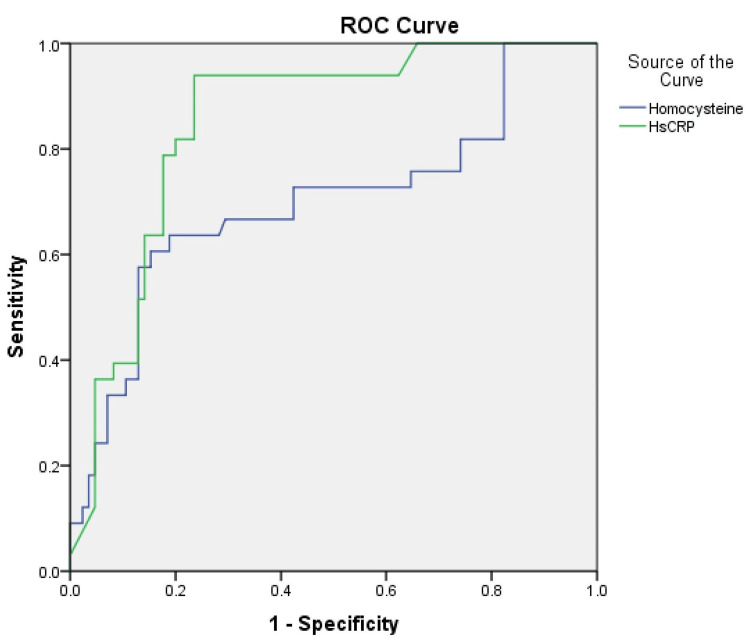
ROC curve for homocysteine and hs-CRP ROC: receiver operating characteristic; hs-CRP: high-sensitivity C-reactive protein

As illustrated in Figure 3, hs-CRP had a slightly higher AUC (0.851 vs. 0.698), suggesting it is a marginally better primary stratification marker for identifying MI. These findings demonstrated that hs-CRP is a superior biomarker compared to Hcy. The higher sensitivity (84.8%) of hs-CRP suggests its usefulness as a screening tool, while its relatively high specificity (76.5%) enhances its reliability in confirming disease presence (Table 7).

**Table 7:** Receiver operating characteristic curve analysis of Older AMI AUC: area under the curve; hs-CRP: high-sensitivity C-reactive protein

Variables	AUC	p-value	95% confidence interval (CI)		Sensitivity	Specificity	Accuracy
			Lower bound	Upper bound			
Homocysteine	0.698	0.001	0.582	0.815	66.7%	57.6%	62.2%
hs-CRP	0.851	0.001	0.779	0.923	84.8%	76.5%	80.7%

## Discussion

The World Health Organization (WHO) has highlighted the growing burden of CVDs in low- and middle-income countries, including India. Epidemiological evidence suggests that CVD in the Indian population progresses more rapidly and tends to manifest at a younger age compared to many other regions [1]. The present study provides insights into the risk factors, clinical profile, and outcomes of young individuals with MI (premature AMI).

Our investigation also revealed that the most commonly identified risk factors for CAD are being male. Among premature patients with AMI, males are predominantly affected, and they constituted 85%, whereas females represented just 15% [17]. Female sex hormones, particularly estrogens, have cardioprotective effects by improving lipid metabolism, endothelial function, and inflammatory pathways, thereby reducing the risk of AMI, especially at younger ages. Consequently, the onset of CVD in females is typically delayed by approximately 7-10 years compared to males [18-20].

Our hospital is a charitable hospital; therefore, many facilities are provided to patients at no cost, including the benefits of all government schemes. It functions as a major healthcare center for the growing semi-urban population in the Karad area. We help bridge the healthcare gap for families living in rural-urban transitional zones. This explains the high proportion of AMI cases from these areas; patients from neighboring towns and rural clusters prioritize our specialized cardiac services and financial accessibility.

Demographic reality and lifestyle contributors

Our hospital functions as a major healthcare center for the surrounding regions, resulting in 70% of our patients coming from semi-urban and rural backgrounds. In this premature AMI cohort, environmental and lifestyle factors heavily dominated over genetics, as only 10% reported a family history of CAD. Physical inactivity (90%), a high red meat diet (85%), and alcohol consumption (55%) were profoundly common.

These findings are consistent with earlier research that shows alcohol consumption, physical inactivity (sedentary lifestyle), and cigarette smoking are key contributors to MI and other long-term cardiovascular conditions. This association suggests a possible biological pathway through which alcohol may influence the development of atherosclerosis [21-23].

Frequent consumption of red meat has been consistently linked to adverse health effects such as CVD and increased all-cause mortality. However, red meat contains both trans and saturated fats. While the fat content of meat has frequently been linked to CHD risk, new research reveals that other meat-related components may contribute more significantly to cardiometabolic disturbances [24].

Also, our study shows a significant relationship between MI and lifestyle factors, including alcohol consumption, physical inactivity, and cigarette smoking. Given the strong link between alcohol consumption, physical inactivity, and smoking, this finding was considered an identifiable novel risk factor in our study. Only 10% of patients with premature AMI had a family history of CAD, which is lower than the percentage found in the control groups.

In our study, AWMI was the most common (50%) kind of ST-segment elevation myocardial infarction (STEMI) observed, followed by IWMI (40%) and LWMI (10%). These findings are similar and consistent with previous studies conducted among patients aged 45 years or younger [21,22].

There was a strong, statistically significant positive correlation between Hcy and hs-CRP in MI patients. The Pearson correlation coefficient (R) was 0.60, with a p-value < 0.001. This indicates that as the level of Hcy increases, the level of hs-CRP also tends to increase, and this relationship is very unlikely to be due to random chance. Hcy demonstrates slightly better diagnostic performance than hs-CRP for identifying AMI in this dataset. hs-CRP levels were significantly higher in both MI groups, particularly in old-age AMI (6.02 ± 4.72 mg/L), suggesting increased systemic inflammation [25-27]. HHcy may contribute to MI through pro-thrombotic and vascular mechanisms. Increased Hcy concentration can lead to platelet activation and thrombus formation, leading to impaired coronary blood flow. Additionally, it reduces nitric oxide, which is a key mediator of endothelial integrity. This impairment can cause endothelial dysfunction and accelerate atherosclerotic progression, and is a key contributor to MI [28].

Increased levels of hs-CRP mean there is more inflammation in the body. This persistent inflammation can further damage the heart muscle, making recovery more challenging [29].

C-reactive protein (CRP) is commonly used to measure inflammation in the body and can help determine the severity of an AMI [16]. Over time, it can increase the risk of complications such as heart failure and recurrent episodes of reduced blood flow to the heart, known as ischemia. Studies suggest that hs-CRP is not only a marker of inflammation but also actively contributes to inflammatory responses and the progression of atherosclerosis [25,30].

Insulin resistance link and age-specific pathophysiology

A pivotal finding of this study is that premature AMI and AMI in older patients present as metabolically distinct phenotypes. Profound, isolated hypertriglyceridemia defines the young cohort (mean TG: 238.00 mg/dL vs. 125.80 mg/dL in older patients). Crucially, the strong correlation between hypertriglyceridemia and glycemic markers uniquely found in young patients exposes subclinical insulin resistance as a primary pathophysiological mechanism. Even with HbA1c in the technically non-diabetic range (mean: 4.51%), this tight TG-glucose correlation exposes a hidden, pre-morbid state of insulin resistance. Older patients with AMI, who instead demonstrated higher classical thrombotic risk via elevated Hcy without the TG-glycemic correlation, represent a separate, age-specific pathophysiology.

​Inflammation-metabolism axis

In all patients with AMI, elevated Hcy and hs-CRP indicate substantial endothelial dysfunction and atherosclerotic progression. However, uniquely in the young cohort, this systemic inflammation (hs-CRP) is tightly coupled with glycemic dysregulation. This suggests that the subclinical metabolic syndrome directly fuels the inflammatory cascade that ultimately leads to premature plaque rupture.

Both MI groups exhibited significantly higher FBS and HbA1c levels than controls. Elevated HbA1c reflects reduced glucose tolerance, which may go unnoticed, subclinical glucose intolerance in young patients with AMI, which can accelerate cardiovascular events by worsening atherogenesis, platelet activation, and endothelial dysfunction [31].

Dyslipidemia is considered one of the main reasons for the higher burden of CAD among South Asians [32], and is characterized by an imbalance in lipid levels, typically involving increased TGs, TC, and low-density lipoprotein cholesterol (LDL-C), along with decreased high-density lipoprotein cholesterol (HDL-C), either separately or in combination [33].

Additionally, our study revealed that young patients with AMI (premature AMI) had much higher TG, followed by increased cholesterol and LDL levels, than healthy controls and old patients with AMI. Patients with premature AMI had significantly lower HDL values when compared with controls [34].

The disproportionate elevation of TGs relative to LDL in the premature AMI group warrants specific attention. Standard cardiovascular risk calculators, including the Framingham Risk Score and pooled cohort equations, assign primary weight to LDL-C and would therefore substantially underestimate the risk in this cohort. The near-normal LDL alongside profoundly elevated VLDL (47.60 ± 23.75 mg/dL) points instead to hepatic overproduction of TG-rich lipoproteins, a hallmark of underlying insulin resistance. This is mechanistically consistent with the strong TG-fasting glucose correlation observed uniquely in the premature AMI group, and reinforces the argument that standard lipid panels without TG-specific interpretation may produce false reassurance in young, apparently low-risk patients.

The pattern of low HDL and high TG levels is referred to as atherogenic dyslipidemia [35]. Several studies demonstrated that dyslipidemia is strongly linked to CVD due to underlying metabolic processes [33]. Our findings support the findings of Sadeghian et al., who also observed increased prevalence of dyslipidemia in individuals with CAD [36]. Previous studies suggest that young patients with AMI have an unfavorable lipid profile, characterized by elevated TGs and reduced HDL-C [37].

## Conclusions

Lifestyle-related risk factors are major drivers of AMI in young adults in semi-urban populations. Premature AMI is characterized by a distinct atherogenic profile dominated by isolated hypertriglyceridemia, chronic inflammation, and subclinical insulin resistance. These metabolic and inflammatory alterations may contribute to endothelial dysfunction, thereby linking adverse lifestyle transitions to accelerated atherogenesis. Managing the rising burden of premature AMI requires shifting from acute care to early, targeted screening for these biomarkers and aggressive lifestyle interventions.
